# Natural course of myopic traction maculopathy and factors influencing progression and visual acuity

**DOI:** 10.1186/s12886-021-02087-y

**Published:** 2021-09-25

**Authors:** Shiwei Li, Tingting Li, Xiangning Wang, Xuan Cai, Bin Lu, Yan Chen, Chang Liu, Qiang Wu

**Affiliations:** Department of Ophthalmology, Shanghai Jiaotong University Affiliated Sixth People’s Hospital, No. 600 Yishan Rd, 200233 Shanghai, China

**Keywords:** High myopia, Myopic traction maculopathy, Natural course, Visual acuity, Optical coherence tomography

## Abstract

**Background:**

To describe the natural course of myopic traction maculopathy (MTM) and determine predictive factors for its progression and visual prognosis.

**Methods:**

This retrospective observational study included 113 MTM patients (113 eyes). Best-corrected visual acuity (BCVA) measurements and optical coherence tomography findings were recorded.

**Results:**

Over a mean follow-up of 38.2 ± 11.1 months, 49 of 113 eyes (43.4 %) progressed. The progression rate of outer schisis prominently located in the fovea or posterior staphyloma was significantly higher than that of outer schisis prominently located in paravascular areas (*P* = 0.0011). MTM with partial posterior vitreous detachment during the follow-up progressed more rapidly than MTM without (*P* = 0.0447). Patients with older age (> 65 years), without domed-shaped macula and with defects in the ellipsoid zone (EZ) had worse BCVA at the last visit (*P* = 0.0416, *P* = 0.0494and *P* = 0.0130). Multiple linear regression analysis showed that BCVA and defects in the EZ at baseline were significantly associated with the final BCVA (*P* < 0.0001 and *P* = < 0.0001, respectively).

**Conclusions:**

MTM has a high possibility for progression. Outer schisis located predominantly in the fovea or posterior staphyloma or with partial posterior vitreous detachment exhibits rapid progression. The integrity of the EZ is related to visual prognosis.

## Background

Myopic traction maculopathy (MTM) refers to a series of pathological changes in the macula in high myopia, including vitreomacular traction, an epimacular membrane, retinoschisis, a lamellar macular hole (LMH) and foveal detachment [1]. Damage to the outer retinal structure or development to foveal detachment and a full-thickness macular hole in the advanced stage can cause visual impairment in MTM, and surgical intervention is recommended to promote anatomical reattachment of the retina and visual recovery [2–5]. Retinoschisis is a major characteristic lesion of MTM, and the incidence of retinoschisis in high myopia with posterior staphyloma is as high as 31.3 % [6]. Retinoschisis can occur in the fovea and extrafovea and in different locations of the intraretinal neural layers due to different dominant pathological factors. However, the mechanism of retinoschisis has not yet been fully elucidated, and many studies have shown that inward and tangential forces produced by partial posterior vitreous detachment (PVD), an epiretinal membrane, arteriosclerosis, and a stiff internal limiting membrane (ILM) and outward traction generated by asynchronous global elongation and posterior staphyloma may play important roles in its pathogenesis [7–11].

Recently, several studies have indicated that the progression rate of MTM varies, that paravascular abnormities and paravascular inner retinoschisis may be associated with the pathogenesis of foveoschisis, and that the severity of retinoschisis in MTM can affect its progression regardless of whether cataract surgery is performed [12–17]. Poor baseline visual acuity is often found in entire macula-involved retinoschisis eyes accompanied by a disruption in the ellipsoid zone (EZ) [16], and progression of MTM can lead to worse visual outcomes in its natural course [15]. However, these studies have focused little on identifying the evolution of retinoschisis prominent in different locations caused by different initiation factors, on the role of inner retinoschisis at paravascular arcades and ILM detachment in the progression of MTM, or on the comparison of the baseline factors affecting visual acuity at follow-up in large series.

Thus, we conducted this study to describe the natural course of MTM, to determine the effects of morphological characteristics of retinoschisis by OCT on MTM progression and to assess the risk factors influencing visual prognosis.

## Methods

### Patients

This retrospective study recruited patients with MTM who initially visited the Department of Ophthalmology of the Sixth People’s Hospital Affiliated to Shanghai Jiao Tong University from June 2014 to March 2018. The inclusion criteria were as follows: (1) MTM diagnosed by OCT, and (2) highly myopic eyes defined as an axis length ≥ 26 mm or a spherical equivalent refractive error ≥-6.00 D. The exclusion criteria were as follows: (1) OCT images with poor quality; (2) a full-thickness macular hole at baseline; (3) a history of vitreoretinal surgery, and (4) other ocular diseases such as glaucoma, retinal vascular diseases or myopic, or age-related macular degenerative diseases. The study adhered to the guidelines of the Helsinki Declaration and had the approval of the Ethics Committee of Sixth People’s Hospital Affiliated to Shanghai Jiao Tong University, Shanghai, China. The study was registered in the Chinese clinical trial registry (http://www.chictr.org.cn/, Registration number: ChiCTR2000038824). All patients signed written informed consent for participation.

### Clinical examinations

All patients were given a comprehensive ocular examination. Best-corrected visual acuity (BCVA) and refractive error were measured by applying a Snellen chart, and BCVA was then converted to logarithmic minimal angle of resolution (logMAR) units for statistical analysis. Axial length was measured by an IOL-Master, and the presence of posterior staphyloma was observed by B-scan ultrasonography. Spectral domain OCT (SD-OCT) (Heidelberg Engineering, Heidelberg, Germany) was performed on the MTM eyes. The SD-OCT scanning protocol consisted of an A-scan through the center of the macula with a length between 9.2 mm and 11.5 mm in the horizontal and vertical direction and 31 B-scans covering an area of 30°×25.0° centered on the fovea at an interval of 256 μm. Two subtypes of retinoschisis were recorded: outer schisis (occurring in the outer plexiform layer) and inner schisis (occurring in the inner plexiform layer and/or ILM detachment). The eyes were classified into 5 groups based on the size and the location of the outer retinoschisis as proposed by Shimada et al. [15]. The central foveal thickness (CFT) was measured and defined as the distance between the hyperreflective band of the ILM and the hyperreflective band of the retinal pigment epithelium through the central fovea and averaging the values measured in the horizontal and vertical A-scans. Then, we determined the most prominent location (upper and lower vascular arcades or their branch vessels, the fovea or the posterior staphyloma) of outer schisis within a diameter of 10 mm centered on the fovea from OCT images, in which the maximum neural thickness (MNT), defined as the distance between the hyperreflective band of the ILM and the hyperreflective band of the retinal pigment epithelium, was measured. Partial PVD, an epimacular membrane, an LMH and the integrity (intact, partially continuous, or absent) of the EZ were examined by OCT. Dome-shaped macula (DSM) is defined as an inward protrusion of the retinal pigment epithelium of the macula ≥ 50 μm in the horizontal or vertical section or both by OCT examination [18].The progression of MTM was characterized as the height of outer schisis increasing by 100 μm, expansion in the extent of outer schisis, or the development of an LMH, FD or a full-thickness macular hole. MTM improvement was termed a reduction in the height or extent of outer schisis unaccompanied with the development of an LMH, FD or a full-thickness macular hole. Cases that did not meet the standard of progression or improvement were treated as stable based on the definition of Shimada et al. [15]. The follow-up time lasted for at least 2 years. BCVA measurements and OCT examinations were performed in all patients at every visit.

### Statistical analysis

Statistical analysis was performed using SAS software version 9.13 (SAS Institute Inc., Chicago, IL). Data are depicted as the mean ± standard deviation (SD). The one-way analysis of variance was used for the comparison of continuous variables, and the chi-square test was used for the statistical analysis of count data. If the sample size in the group was < 5, Fisher’s exact test was performed. If the data did not conform to a normal distribution, the Kruskal-Wallis test was performed. We determined the factors influencing BCVA at the last visit using multivariate regression analysis. When *P* was < 0.05, the difference was considered statistically significant.

## Results

### Baseline characteristics

We enrolled 113 patients (113eyes) with MTM in this study, and the mean follow-up time was 38.2 ± 11.1 months. The baseline characteristics of all patients are listed in Table 1.

**Table 1:** Demographics and baseline characteristics of patients with myopic traction maculopathy

Variable	Value
No. of subjects	113
No. of eyes	113
Sex, male/female	38/75
Age (years),mean ± SD (range)	65.1 ± 9.6 (34 to 83)
Refractive error (D), mean ± SD (range) (phakic eyes, *n* = 66)	-12.6 ± 4.8(-6.0 to -23.0)
Axial length (mm), mean ± SD (range)	29.3 ± 1.83(26.00 to 36.03)
BCVA in Snellen equivalent	20/50 ± 20/50(20/1000-20/20)
BCVA in logMAR, mean ± SD	0.42 ± 0.39
Posterior staphyloma (n)	88
Pseudophakic eyes(n)	47
Follow-up duration (months) Mean ± SD (range)	38.2 ± 11.1(24 to 71)
Schisis group (n)	
S0	8
S1	24
S2	9
S3	46
S4	26
The most prominent location of the outer schisis (n)^a^	
Paravascuslar	43
Fovea or posterior staphyloma	62
Schisis subtype (n)^a^	
Outer schisis	67
Outer and inner schisis	38
Partial PVD (n)	33
Epimacular membrane (n)	52
LMH (n)	25
CFT(µm)	275.4 ± 124.6(112–732.5)
MNT(µm)	413.4 ± 116.7(206–711)
DSM (n)	16
Defect of EZ(n)	21

*SD* standard deviation, *D *diopter, *LogMAR* logarithm of the minimal angle of resolution, *PVD* posterior vitreous detachment, *LMH* lamellar macular hole, *EZ* ellipsoid zone, *CFT* central foveal thickness, *DSM* dome-shaped macula

^a^Eight eyes with S0 stage were excluded

### Changes in morphological characteristics by OCT

**Fig. 1:**
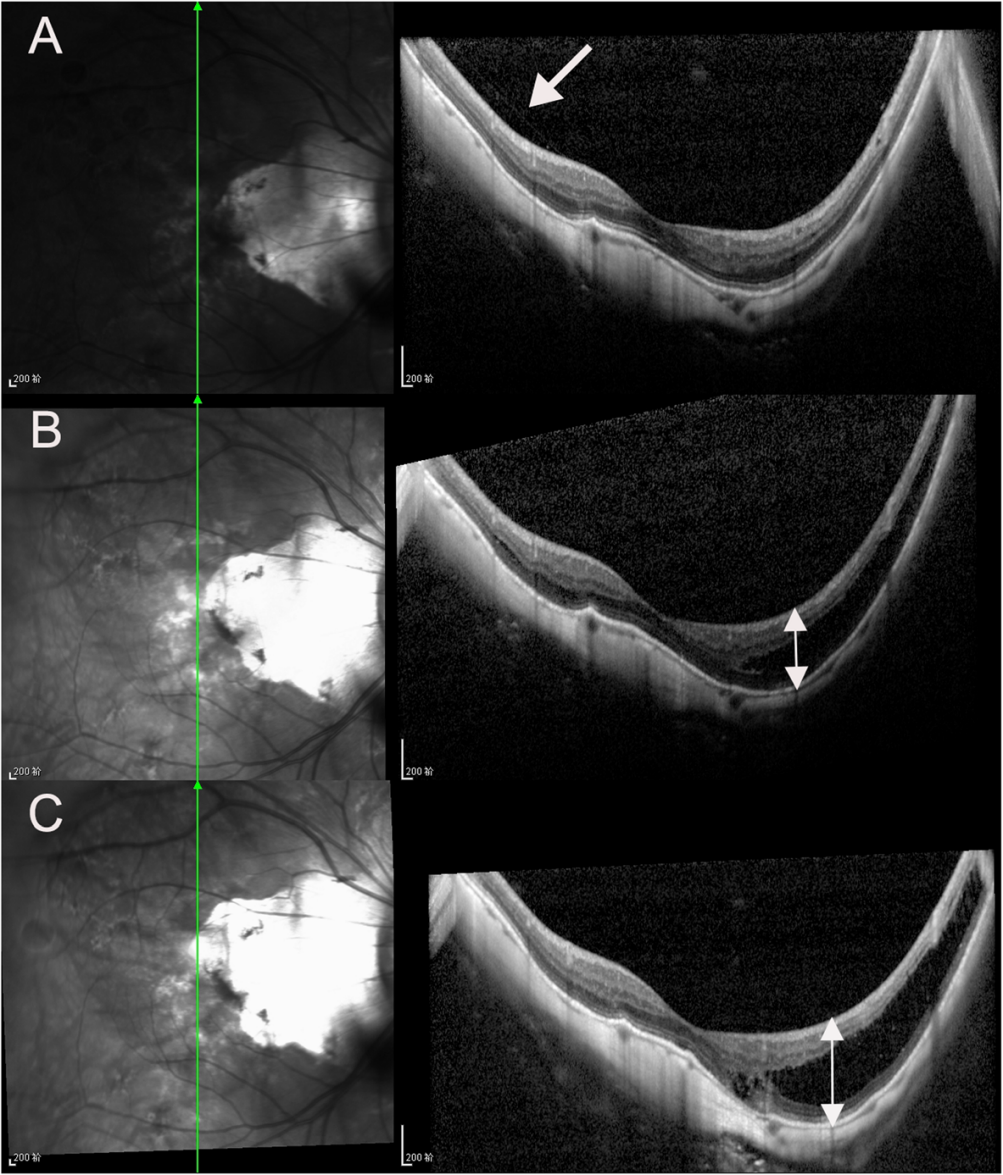
Infrared fundus and OCT images of the right eye of a 73-year-old man with MTM that progressed during the follow-up period in vertical scan. At baseline, the case had S0 MTM with partial posterior vitreous detachment (PVD) (white arrow), the ellipsoid zone (EZ) was intact, and BCVA was 20/63 (**A**). Tirty-six months later, S3 MTM with outer schisis occurred in the location of posterior staphyloma, and maximum neural thickness (MNT) (white double arrow)was 399 μm, and BCVA was 20/63 (**B**). At 68 months after the first visit, the height of schisis increased, and MNT was 525 μm (white double arrow), EZ was partially continuous, and BCVA was 20/100 (**C**)

**Fig. 2:**
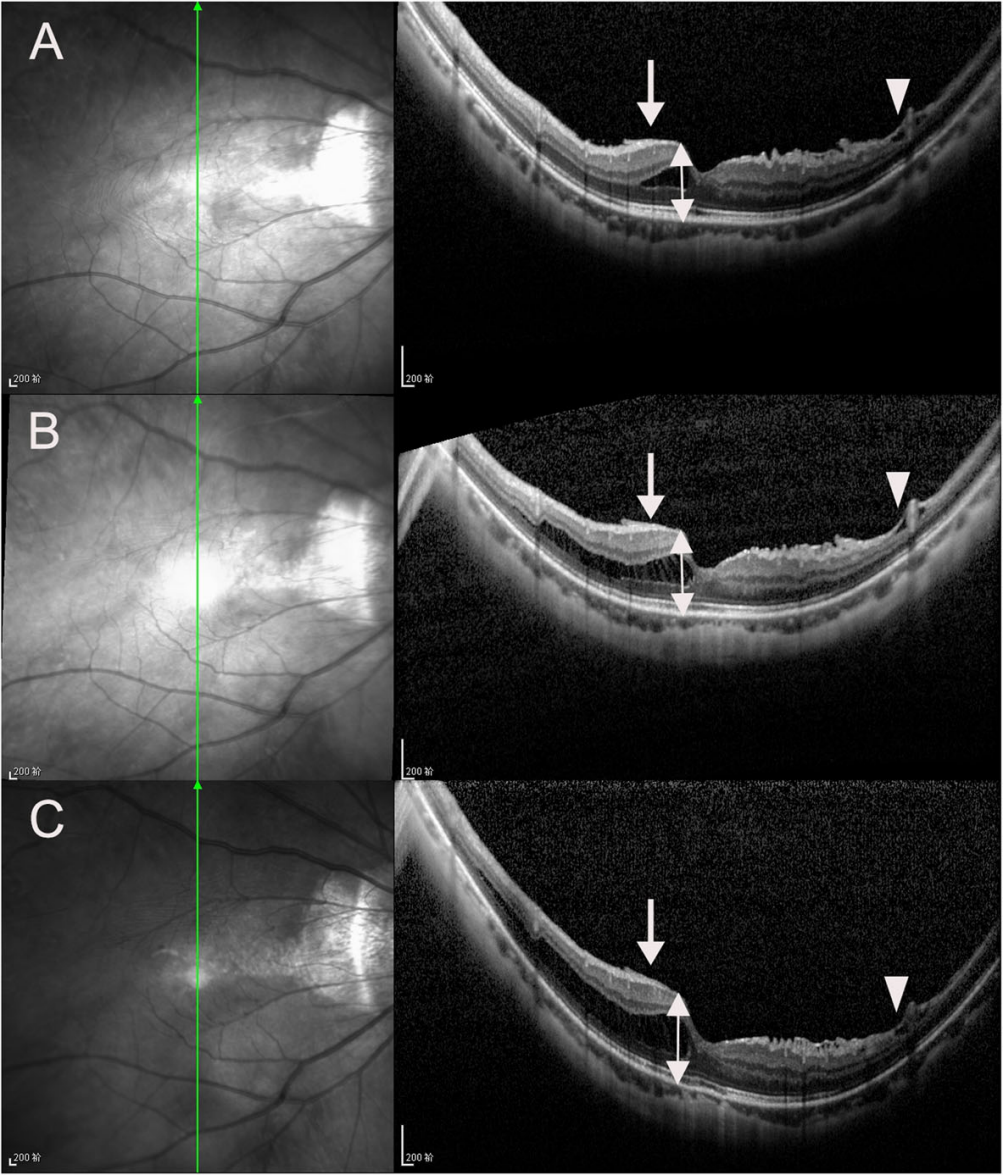
Infrared fundus and OCT images of the right eye of a 51-year-old woman with MTM that progressed during the follow-up period in vertical scan. At baseline, the case had S2 MTM with outer schisis in fovea, inner schisisin upper vascular arcades (white arrow head) and epimacular membrane (white arrow), MNT (white double arrow) was 361 μm, and BCVA was 20/32 (**A**). Twenty-seven months later, S3 MTM occurred, MNT (white double arrow) was 399 μm, and BCVA decreased to 20/40 (**B**). At 41 months after the first visit, the extent of schisis enlarged, the height of schisis increased, MNT (white double arrow) was 431 μm, and BCVA was 20/40. EZ was intact during the follow-up period (**C**)

**Fig. 3:**
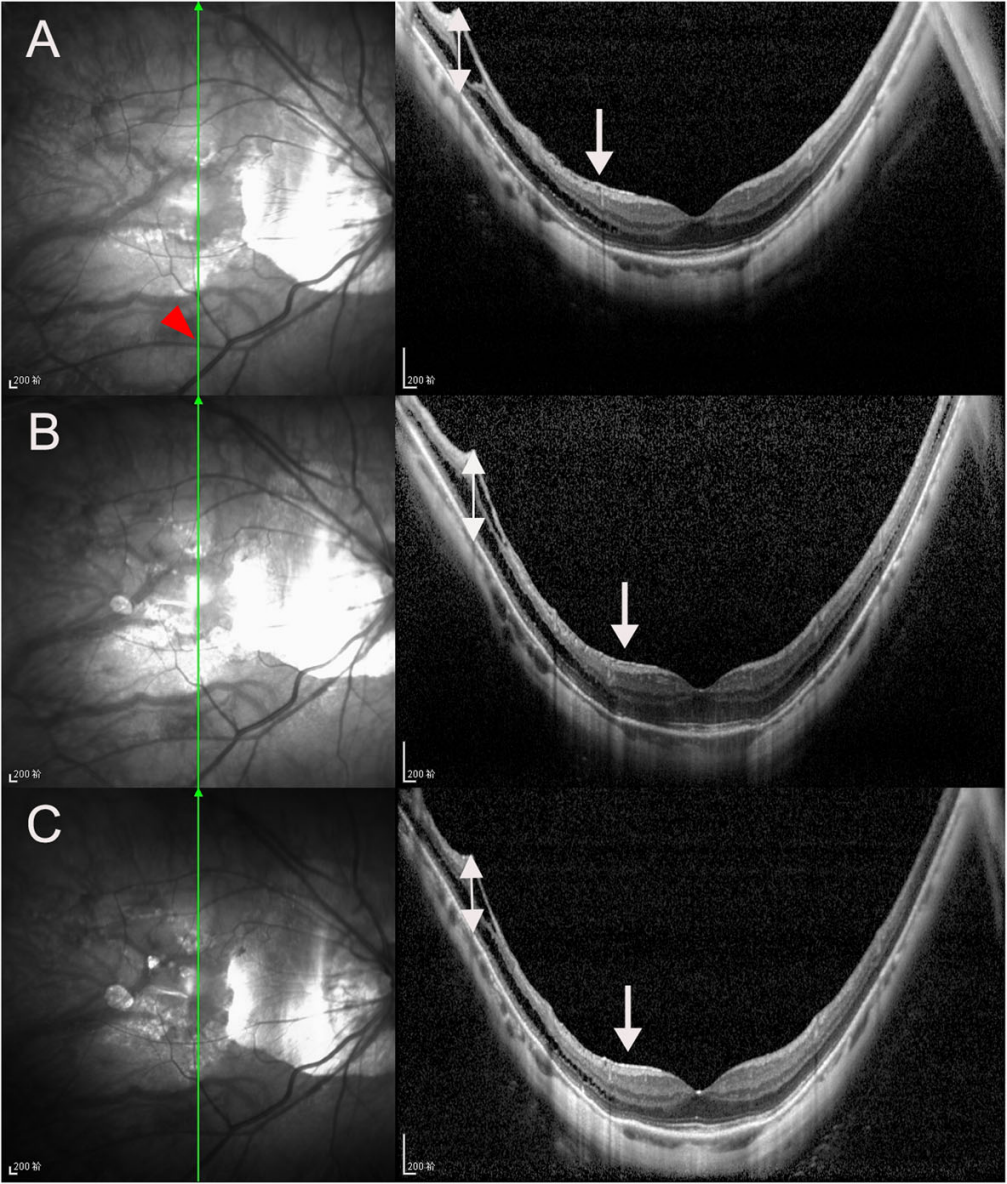
Infrared fundus and OCT images of the right eye of a 57-year-old man with stable MTM during the follow-up period in vertical scan. At baseline, the case had S3 MTM with outer schisis prominently located in the inferior paravascular area (red arrow head) and inner schisis in the inferior paravascular area, and with epimacular membrane (white arrow), MNT (white double arrow) was 390 μm (**A**). After 53 months, MNT (white double arrow) was 406 μm (**B**). At 67 months after the first visit, MNT (white double arrow) was 405 μm, EZ was intact and BCVA was 20/32 during the follow-up period (**C**)

**Fig. 4:**
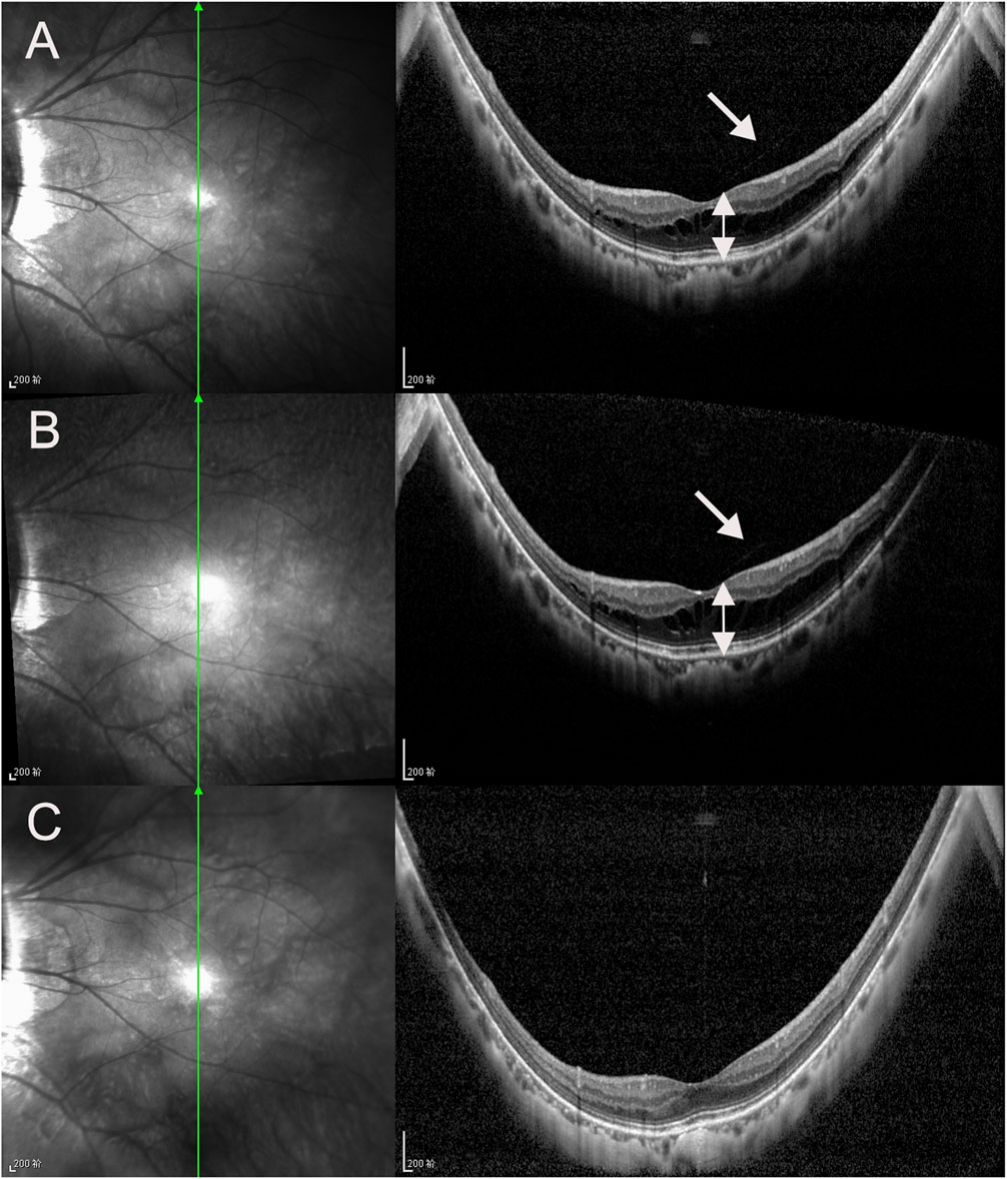
Infrared fundus and OCT images of the left eye of a 57-year-old woman with MTM for improvement during the follow-up period in vertical scan. At baseline, the case had S3 MTM with outer schisis prominently located in the fovea (also in posterior staphyloma) and partial PVD (white arrow), MNT (white double arrow) were 308 μm, and BCVA was 20/50 (**A**). After 10 months, MNT (white double arrow) were 333 μm, and BCVA was 20/50 (**B**). At 37 months after the first visit, partial PVD detached from the fovea, the outer schisis disappeared in vertical scan, and BCVA improved to 20/25. EZ was intact during the follow-up period (**C**)

**Fig. 5:**
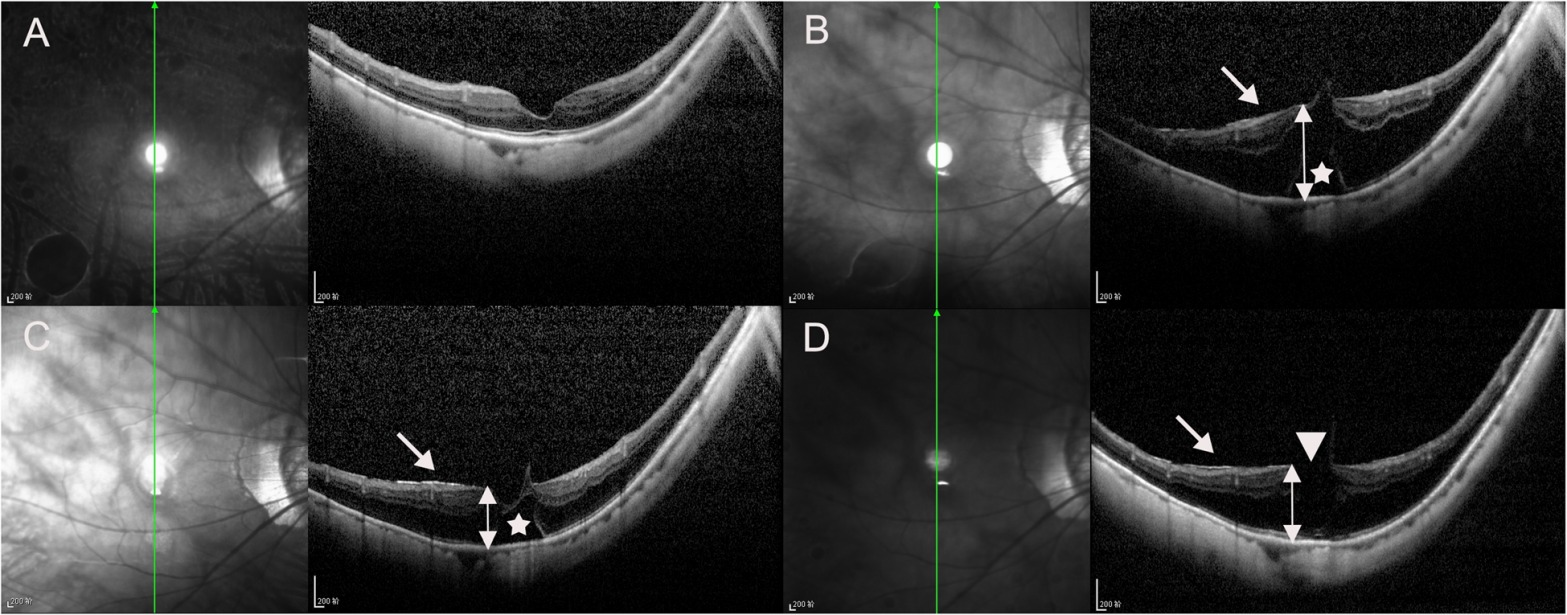
Infrared fundus and OCT images of the right eye of an 80-year-old woman with MTM for progression during the follow-up period in vertical scan. At baseline, the case had S0 MTM, the ellipsoid zone was intact on the fovea, and BCVA was 20/50 (**A**). After 39 months, S4 retinoschisis with an epimacular membrane (white arrow) was seen and a foveal detachment (FD) (white asterisk) developed, MNT (white double arrow) were 665 μm and BCVA declined to 20/200 (**B**). At 44 months after the first visit, the height of outer retinoschisis decreased, MNT (white double arrow) was 368 μm, and BCVA was still 20/200 (**C**). At 67 months after the first visit, the FD resolved spontaneously, a lamellar macular hole (white triangle) developed, the height and of outer retinoschisis increased, MNT (white double arrow) was 496 μm, while the extent of the outer retinoschisis decreased, and BCVA declined to 20/400 (**D**)

There were 5, 23, 8, 42 and 35 eyes in stages S0, S1, S2, S3, and S4 of MTM, respectively, at the last visit. In the follow-up period, 49 eyes (43.4 %) progressed (Figs. 1 and 2), 45 eyes (39.8 %) remained stable (Fig. 3), and 19 eyes (16.8 %) experienced improvement (Fig. 4). A full-thickness macular hole was found in 1 eye, an LMH developed in 3 eyes, and FD developed in 6 eyes (Fig. 5). At the last visit, ILM detachment was disrupted or disappeared in 4 eyes that did not experience progression.

### Risk factors for progression

We divided the patients into three groups, progressive, stable and improved, to explore the possible risk factors affecting progression (Table 2). When we determined the influence of the subgroup, subtype and location of retinoschisis on progression, 8 eyes with S0 at baseline were excluded. The progression rate in eyes with outer schisis most prominently located in the fovea or staphyloma (51.6 %) was significantly higher than that in eyes with outer schisis located in the vascular arcades (20.9 %) (*P* = 0.0011). Eyes with partial PVD during the follow-up had a higher proportion of progression (60.5 %) than those without PVD (32.9 %) (*P* = 0.0447). In contrast, age, axial length, presence of posterior staphyloma, epimacular membrane, subgroup, subtype of schisis or DSM were not different among the three groups (*P* > 0.05).

**Table 2:** Factors associated with evolution of myopic traction maculopathy

Factor	Progressed	Stable	Improved	*P value*
Age (years), mean ± SD	64.7 ± 10.2	66.1 ± 9.3	62.4 ± 8.0	0.2267*
Refractive error (D), mean ± SD	-11.8 ± 4.4	-12.9 ± 4.4	-13.8 ± 6.2	0.3233*
Axial length (mm), mean ± SD	29.3 ± 1.4	29.3 ± 2.0	29.3 ± 2.4	0.9436*
Posterior staphyloma				0.8889**
Present	39	34	15	
Absent	10	11	4	
Schisis subgroup (n)^a^				0.2916**
S1	8	10	6	
S2	3	5	1	
S3	20	16	10	
S4	10	14	2	
Schisis subtype (n)^a^				0.4098**
Outer schisis	29	26	12	
Outer and inner schisis	12	19	7	
The most prominent location of outer schisis (n)^*^				0.0011**
Paravascuslar	9	22	12	
Fovea or posterior staphyloma	32	23	7	
Epimacular membrane during a follow-up (n)				0.0636**
Present	27	19	6	
Absent	22	26	13	
Partial PVD during a follow-up(n)				0.0447**
Present	26	10	7	
Absent	23	35	12	
LMH (n)				0.4645**
Present	11	12	2	
Absent	38	33	17	
DSM (n)				0.1175
Present	4	8	4	
Absent	45	37	15	

*SD* standard deviation, *PVD* posterior vitreous detachment, *LMH* lamellar macular hole, *DSM* dome-shaped macula

^a^Eight eyes with S0 stage were excluded

*Significant difference among group means for age and axial length via one-way analysis of variance(*P* < 0.05)

**Significant difference among three groups for the other factors via chi-square test (*P* < 0.05)

### Changes in BCVA and predictive factors for visual acuity

LogMAR BCVA at the last follow-up was worse than that at baseline in all patients (*P* = 0.0372), in patients who were older than 65 years, in patients without DSM, and in patients with defects in the EZ (*P* = 0.0416, *P* = 0.0494, and *P* = 0.0130, respectively) (Table 3). In the multiple linear regression analysis, logMAR BCVA (*P* < 0.0001) and a defect in the EZ at baseline (*P* < 0.0001) were significantly correlated with logMAR BCVA at the last follow-up (Table 4).

**Table 3:** Comparison of visual acuity at first and last visits with different baseline characteristics

Characteristics	BCVA at First Visit	BCVA at Last Visit	P value*
	Mean ± SD, logMAR	Mean ± SD, logMAR	
All patients	0.42 ± 0.39	0.58 ± 0.53	0.0372
Age			
≥65years	0.49 ± 0.39	0.71 ± 0.56	0.0416
< 65years	0.35 ± 0.37	0.45 ± 0.48	0.2603
Axial length			
>30mm	0.45 ± 0.44	0.65 ± 0.63	0.2740
<30mm	0.40 ± 0.36	0.54 ± 0.48	0.0702
Posterior staphyloma			
Present	0.44 ± 0.39	0.63 ± 0.56	0.0565
Absent	0.32 ± 0.35	0.40 ± 0.40	0.3444
Lamellar macular hole			
Present	0.72 ± 0.49	0.99 ± 0.62	0.1295
Absent	0.33 ± 0.30	0.46 ± 0.45	0.0643
CFT			
≥300 μm	0.58 ± 0.44	0.79 ± 0.60	0.1859
<300 μm	0.34 ± 0.33	0.48 ± 0.48	0.0678
DSM			
Present	0.46 ± 0.47	0.64 ± 0.62	0.3931
Absent	0.41 ± 0.37	0.57 ± 0.52	0.0494
EZ			
Intact or partially continuous	0.34 ± 0.31	0.44 ± 0.39	0.1118
Defect	0.72 ± 0.52	1.20 ± 0.64	0.0130

*LogMAR* logarithm of the minimal angle of resolution, *CFT* central foveal thickness, *DSM* dome-shaped macula; *EZ* ellipsoid zone

*Significant difference between the group means at the first and last visit via Kruskal-Wallis test (*P* < 0.05)

**Table 4:** Multiple linear regression analysis of baseline characteristics associated with visual acuity at final visit

Characteristics	Standardized estimate	P value*
Age (years)	0.0445	0.4447
Axial Length (mm)	-0.0051	0.9296
BCVA (LogMAR) at first visit	0.6325	< 0.0001
Posterior Staphyloma	0.0523	0.3338
LMH	0.0631	0.3729
Schisis Subgroup	0.0039	0.9468
Schisis Subtype	-0.0020	0.9705
CFT(µm)	0.0438	0.5245
DSM	0.0304	0.5921
Defect of EZ	0.2718	< 0.0001

*LogMAR* logarithm of the minimal angle of resolution, *CFT* central foveal thickness, *DSM* dome−shaped macula, *EZ* ellipsoid zone

*Significant difference via multiple linear regression analysis (*P* < 0.05)

## Discussion

Our study found that 49/113 eyes (43.4 %) with MTM progressed over the 2-year follow-up period, and eyes with retinoschisis prominently located in the fovea or posterior staphyloma were prone to progression. Vitreoretinal traction may be one of the risk factors for progression, and the integrity of the EZ was considered to be a main factor affecting visual prognosis.

The progression rate of MTM or retinoschisis varies, ranging between 11.6 and 68.9 %, according to different standards [14, 15]. Gaucher et al. [14] found that 68.9 % (20/29 eyes) of the eyes with foveoschsis progressed with enlargement of retinal cleavage and/or visual decline during a mean follow-up period of 31.2 months. In our retrospective study, 41/105eyes (39.0 %) with MTM progressed based on the criteria of morphological changes of MTM described by Shimada et al. [15], even when  8 S0 eyes were excluded at baseline, which was much higher than that reported by Shimada et al. [15] (11.6 %). The different progression rates may be related to the difference in age, the status of vitreomacular traction, the involved location and area of schisis or the follow-up period in these studies.

Our results showed that eyes with outer schisis prominently located in the fovea or posterior staphyloma had a higher risk of progression than those in which outer schisis located in the vascular areas. The mechanism of retinoschisis is complicated, and the growth of the eyeball is considered an initial factor in the pathogenesis of foveoschisis [19]. Shinohara et al. [20] suggested that posterior staphyloma may act as the main cause of retinoschisis located within the area of the posterior staphyloma by ultrawide-field swept-source OCT, and outer and inner retinoschisis located in vascular arcades may be caused mainly by vitreous adhesion in the blood vessels of the retina and the tractional force of the retinal arterioles [8, 21]. However, after reaching the loose outer retina through the transmission of intraretinal tissues, the inner traction may become weak, and the inner retina may play a “shock-absorbing”-like role, while outward traction is direct and persistent, which may be the reason why outer retinoschisis at the blood vessel develops more slowly than that in the fovea. In our series, almost all cases of inner schisis were confined to the paravascular area adjacent to the superior or inferior temporal vascular arcades, which may result from tangential traction of the retinal arterioles. The presence of inner schisis did not significantly promote the progression of outer schisis in different locations regardless of the presence of ILM detachment, revealing that the retina itself may play a secondary role in the occurrence and development of outer schisis. In this study, MTM with partial PVD during follow-up was more likely to progress. Shinohara et al. [20] also proposed that the posterior vitreous extensively adhered to the retinal surface, exerting persistent inward traction in eyes of retinoschisis, which may contribute to the development of retinoschisis without posterior staphyloma. Additionally, we found that the stage of schisis had no significant influence on the risk of progression. Shimada et al. [15] and Cheng et al. [16] suggested that the status of S4 eyes was unstable and had a high risk for deterioration. Cai et al. [17] also believed that as long as the schisis involved the fovea, regardless of whether it extended to the entire macula, the possibility of progression was relatively high. Therefore, attention should be paid to each stage of MTM, especially in eyes with vitreomacular adhesion. Meanwhile, we observed that eyes in which ILM detachment was disrupted or disappeared did not progress. In foveoschisis, Müller cells and astrocytes proliferate to produce tangential stress to separate the intraretinal tissues [10]. Once ILM detachment is disrupted, tangential stress is released, and the splitting cavity may shrink or even completely disappear. In addition, recent studied have shown that extrafoveal retinoschisis is more common in highly myopic eyes with dome-shaped macula (DSM) than foveal retinoschisis [22, 23], and DSM may reduce the traction on the fovea, acting as a macular buckle [18], whereas, in our study, there was no significant difference in the progression rate of MTM between the DSM group and the non-DSM group, and the presence of DSM did not seem to delay the progression of MTM, the possible reason may be that the existence of DSM was not enough to completely offset or resist the inward or tangential traction force acting on the retina or the shape of the posterior staphyloma changed during follow-up period in our cases.

In this study, patients who were older than 65 years, who had not a DSM, and who had EZ defects at the first visit had poor final BCVA, and further multiple linear regression analysis showed that first-visit visual acuity and EZ defects are factors affecting visual prognosis. The interruption or absence of reflection EZ in the outer retina of retinoschisis may represent the abnormal energy metabolism of the elongated mitochondria of the photoreceptors, which means that visual function is impaired. Damage to EZ is not uncommon in patients with retinoschisis. Sayanagi et al. [24, 25] reported that the incidence of EZ defects in foveoschisis is between 29 and 38 %. Studies on the influencing factors of vision recovery after vitrectomy for foveoschisis have confirmed that the recovery of the EZ has a significant correlation with postoperative vision recovery [26, 27]. Cheng et al. [16] reported that 6/14 (42.9 %) patients with retinoschisis who had ≥ 2 lines of vision loss in their natural courses had EZ disruption at the first visit. We speculate that some elderly patients had a long course of schisis, and the baseline EZ was not intact or even absent, the photoreceptors were damaged severely over time, which subsequently caused poor vision. Even in pseudophakic eyes, the improvement in their visual function was not satisfactory after cataract surgery. In addition, Zhu and her colleagues [23] proposed that DSM brought better visual acuity after cataract surgery in highly myopic eyes. BCVA of DSM eyes at the final visit was not significantly different from that at the first visit in our study, the presence of DSM may reduce the risk of the deterioration of visual acuity, nevertheless, EZ defect was still the leading factor of visual impairment in our study.

Our study has some limitations. First, the occurrence and development of cataracts in some elderly patients who experienced a long follow-up time inevitably affected visual acuity, and an electrophysiological examination may be required as a good supplement to evaluate visual function and prognosis. Second, the exertion of centrifugal vertical and tangential forces may cause an increase in the height and expansion of the extent of retinoschisis, respectively. In future studies, if we can increase the sample size, quantify the extent of the schisis, supplement the criteria for the progression of MTM, and further explore the equivalence of the impact of the expansion of the extent and the increase in height of the schisis on the progression of MTM, it may be of some significance to investigate its pathogenesis.

## Conclusions

In summary, MTM had a high progression rate during the follow-up. MTM progression was related to the location of retinoschisis. Vitreomacular traction may play an important role in the natural course of MTM. Defects in the EZ have a negative influence on visual prognosis, which may provide some clues to the long-term evolution and pathogenesis of MTM.

## Data Availability

The data used and analyzed during the current study are available from the corresponding author on reasonable request. The data supporting our findings can also be found in the Chinese clinical trial registry (http://www.chictr.org.cn/, Registration number: ChiCTR2000038824).

## Abbreviations

MTM: Myopic traction maculopathy
EZ: Ellipsoid zone
LMH: Lamellar macular hole
PVD: Posterior vitreous detachment
ILM: Internal limiting membrane
BCVA: Best-corrected visual acuity
Log MAR: Logarithmic minimal angle of resolution
SD-OCT: Spectral domain OCT
CFT: Central foveal thickness
MNT: Maximum neural thickness
SD: Standard deviation
